# The combination of sarcopenia and biochemical factors can predict the survival of hepatocellular carcinoma patients receiving transarterial chemoembolization

**DOI:** 10.3389/fonc.2022.1005571

**Published:** 2022-09-29

**Authors:** Tzu-Ping Chien, Song-Fong Huang, Wen-Hui Chan, Kuang-Tse Pan, Ming-Chin Yu, Wei-Chen Lee, Hsin-I Tsai, Po-Ting Lin, Hsing-Yu Chen, Jui-Hsuan Chen, Chao-Wei Lee

**Affiliations:** ^1^ Division of General Surgery, Department of Surgery, Linkou Chang Gung Memorial Hospital, Taoyuan, Taiwan; ^2^ Division of General Surgery, Department of Surgery, New Taipei Municipal Tu-Cheng Hospital (Built and Operated by Chang Gung Medical Foundation), New Taipei City, Taiwan; ^3^ College of Medicine, Chang Gung University, Taoyuan, Taiwan; ^4^ Department of Medical Imaging and Intervention, Linkou Chang Gung Memorial Hospital, Taoyuan, Taiwan; ^5^ Department of Anesthesiology, Linkou Chang Gung Memorial Hospital, Taoyuan, Taiwan; ^6^ Graduate Institute of Clinical Medical Sciences, Chang Gung University, Taoyuan, Taiwan; ^7^ Department of Gastroenterology and Hepatology, Linkou Chang Gung Memorial Hospital, Taoyuan, Taiwan; ^8^ Division of Chinese Internal Medicine, Center for Traditional Chinese Medicine, Taoyuan Chang Gung Memorial Hospital, Taoyuan, Taiwan; ^9^ School of Traditional Chinese Medicine, College of Medicine, Chang Gung University, Taoyuan, Taiwan; ^10^ Department of Nursing, Linkou Chang Gung Memorial Hospital, Taoyuan, Taiwan

**Keywords:** hepatocellular carcinoma, sarcopenia, transarterial chemoembolization (TACE), survival (MeSH), biochemical factors

## Abstract

**Background:**

Transarterial chemoembolization(TACE) is the suggested treatment for hepatocellular carcinoma (HCC) not amenable to curative treatments. We investigated the role of sarcopenia on overall survival in HCC patients receiving TACE and proposed a new prognostic scoring system incorporating sarcopenia.

**Materials and methods:**

We retrospectively analyzed 260 HCC patients who received TACE between 2010 and 2015. Total psoas muscle was measured on a cross-sectional CT image before the first TACE session. Sarcopenia was defined by the pre-determined sex-specific cutoff value. We assessed the impact of sarcopenia and other biochemical factors on the overall survival and compared the new scoring system with other prognostic scoring systems.

**Results:**

One hundred and thirty patients (50%) were classified as sarcopenia before the first TACE. They were older with a higher male tendency and a significantly lower body mass index (BMI). Cox regression multivariate analysis demonstrated that sarcopenia, multiple tumors, maximal tumor diameter≥ 5cm, major venous thrombosis, sarcopenia, AFP ≥ 200 ng/ml, and albumin<3.5mg/dL were independent poor prognostic factors for overall survival in HCC patients receiving TACE. Our scoring system comprising these factors outperformed other major scoring systems in terms of predicting survival after TACE.

**Conclusion:**

The current study demonstrated that sarcopenia was an independent prognostic factor for HCC undergoing TACE therapy. Our newly developed scoring system could effectively predict patient survival after TACE. Physicians could, based on the current score model, carefully select candidate patients for TACE treatment in order to optimize their survival. Further studies are warranted to validate our findings.

## Introduction

Hepatocellular carcinoma (HCC) is the sixth most common cancer worldwide and the third common cause of cancer deaths in 2020 (1). The Barcelona Clinic Liver Cancer (BCLC) staging system is generally applied in clinical practice to guide the treatment plans for HCC. Curative treatments such as radiofrequency ablation (RFA), surgical resection and liver transplantation are indicated for very early- or early-stage HCC and can achieve promising outcome. However, for intermediate stage HCC, such as those with multiple nodules or impaired liver function, curative therapy is not always applicable and transarterial chemoembolization (TACE) is the recommended treatment modality (2, 3). In addition to intermediate stage, some selected advanced stage HCC patients may also benefit from TACE (4). Clinically, TACE is repeated either periodically or as demand and withheld at disease progression or severely impaired liver function. It could result in an estimated average median overall survival of 26 to 32 months (2, 5). However, patients with intermediate or advanced stage HCC present with a broad range of tumor biology, tumor burdens, liver function, and comorbidities and may not all benefit from the TACE treatment. This explains the wide range of survival outcome reported in different literatures (6). As a result, efforts have been made to evaluate the efficacy of TACE either before or at the beginning of TACE sessions. Many prognostic models were developed to assess the efficiency of TACE, such as the Hepatoma Arterial Embolization Prognostic score (HAP) score, Assessment for Retreatment with TACE (ART) score, Six-and-Twelve prognostic score, Up-to-Seven score, and albumin-bilirubin (ALBI) grade (7–15). However, most of these models or systems consider only tumor and/or liver factors; few scoring systems to date had incorporated general status of patients such as body composition into consideration.

Sarcopenia, for example, is a marker of body composition that raises researcher’s attention. Sarcopenia is characterized by loss of muscle mass, strength, and function (16, 17). The development of sarcopenia may be related to age, physical activity, nutrition, and diseases such as cancer or chronic inflammation (16, 18, 19). Patients with sarcopenia are particularly vulnerable in the setting of major physiologic stress including major surgery and critical illness (18, 20). In addition to adverse surgical outcome, studies have demonstrated that low skeletal muscle mass was also associated with worse short-term and long-term clinical outcomes in several cancers, including gastric, esophageal, pancreatic, lung, bladder, breast, and colon cancers (21–29). For HCC per se, sarcopenia was reported to be an independent poor prognostic factor for HCC undergoing liver resection (30–32). Both the recurrence-free and overall survivals were compromised in HCC patients with a low skeletal muscle mass. Sarcopenia, as a result, could be an important prognostic factor that should be taken into consideration prior to HCC resection. Nevertheless, the influence of sarcopenia in HCC patients undergoing TACE treatment has not been largely examined (33–35). The aim of the current study was thus to investigate the role of sarcopenia on overall survival in HCC patients receiving TACE and to propose a new prognostic scoring system incorporating sarcopenia.

## Material and methods

### Patients and study design

**Figure 1:**
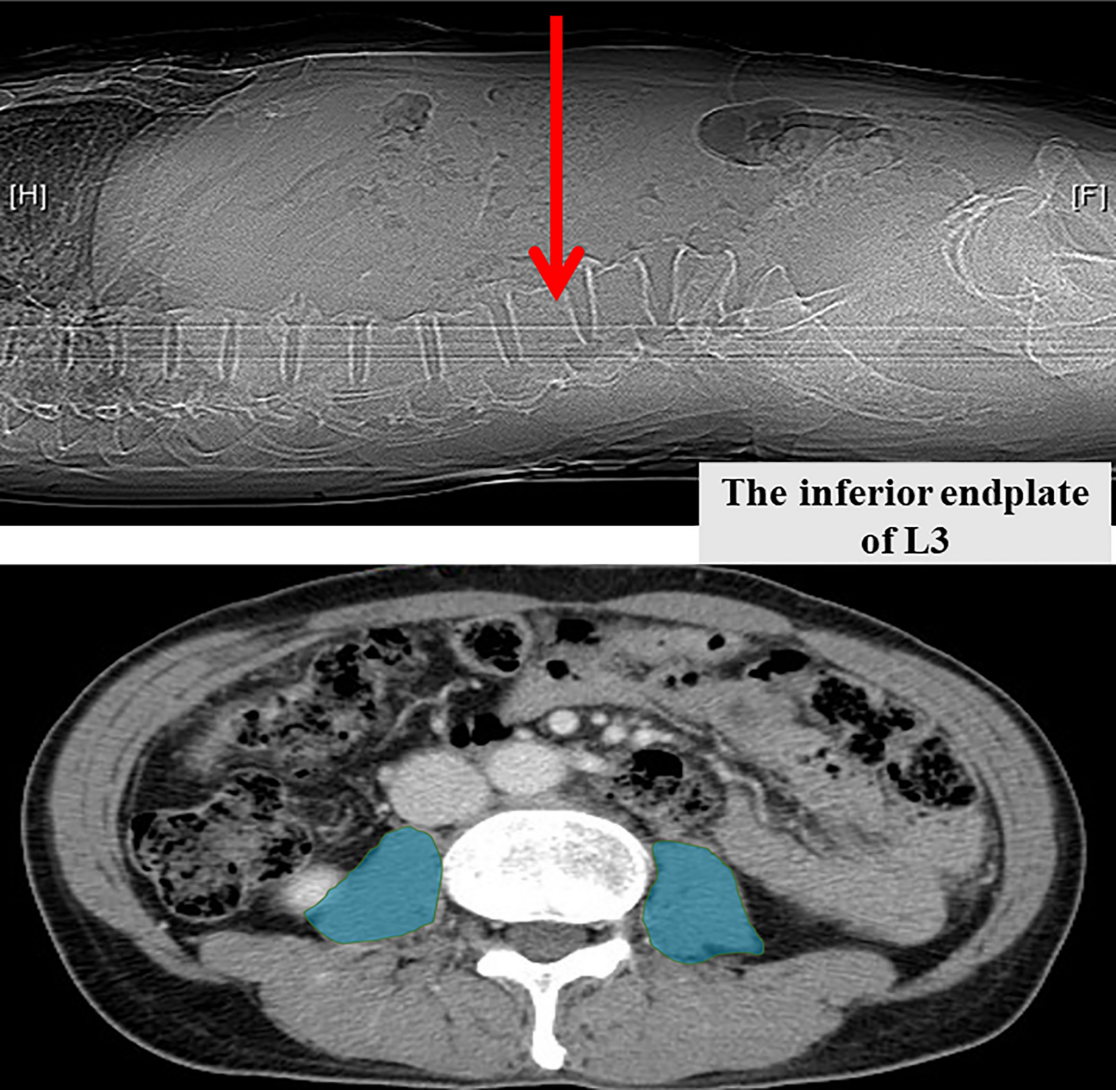
Measurement of bilateral psoas muscle area at the inferior endplate of the third lumbar vertebra.

We conducted a retrospective review of HCC patients who received TACE as primary treatment between January 2010 and August 2015 in Linkou Chang Gung memorial hospital. Patients who received either radiofrequency ablation (RFA), liver resection, or liver transplantation after TACE were excluded from the current study. A total of 260 patients were finally enrolled. The abdominal computed tomography (CT) within 30 days before the first TACE was retrieved and reviewed. A multidetector CT scanner (Aquilion ONE, Toshiba Medical Systems, Tochigi, Japan) was used to obtain the detailed image. CT technical parameters included 120 kV (tube voltage), 0.5 mm 320 row (detector configuration), tube current modulation, 0.35 sec/rotation (gantry rotation), and 16mm reconstruction thickness. The cross-sectional area of psoas muscles at the level of the inferior endplate of the third lumbar vertebrae were obtained from the CT scan. The attenuation between –30 HU to 150 HU were applied to identify the muscle mass (36). The areas (cm^2^) of bilateral psoas muscles were measured by a picture processing and analyzing software, FIJI, which is an open source image processing package (**Figure 1**) (37, 38). The areas were further normalized by the patients’ height squared (cm^2^/m^2^) (39, 40). The sex-specific cut-off value for sarcopenia is defined as 6.36 cm2/m2 for men and 3.92 cm2/m2 for women (41). Clinical data including patient age, body mass index(BMI), AJCC tumor stage, viral hepatitis markers, tumor size, tumor number, hemogram, liver function tests, and survival outcome were recorded and analyzed. Tumor staging was based on the 8th edition of American Joint Committee on Cancer Tumor-node-metastasis (AJCC TNM) staging system for HCC (42). The primary study endpoint was the determination of the impact of sarcopenia on overall survival in HCC patients receiving TACE, and the secondary endpoint was the development a scoring system prognostic of 3- and 5-year survivals after TACE. This study was approved by the Institutional Review Board of the Chang Gung memorial hospital, No. 202102599B0.

### Transarterial chemoembolization (TACE)

The conventional TACE were conducted by dedicated interventional radiologists and was repeated every 2-3 months based on tumor response and liver functions (8, 43, 44). The mean session of TACE was 5.6 and median was 4 sessions per patient in the current study. Before each session of TACE, adequate intravenous hydration was administered and the patient was put on *nil per os* (NPO). Vascular access was established *via* common femoral artery puncture. A 4-French catheter was used to cannulate the visceral arteries. Angiography was performed to identify the tumors and their feeding arteries, and the patency of portal vein was also confirmed. Chemoembolization employed a mixture of doxorubicin hydrochloride (75 mg/m^2^ body surface area) with 10 mL of lipiodol (Lipiodol Ultra-fluide, Guerbet, Aulnay-sous-Bois, France). Gelatin sponge pledges were then injected to ensure stagnation of the blood flow in the embolized vessel (45). After TACE, all patients were hospitalized and monitored for at least 24 hours. Patients were then followed at a 3 to 4-month interval with their tumor markers and liver biochemical parameters examined. Serial imaging studies including abdominal CT will be performed regularly as well. The TACE procedures would be withheld when there were compromised liver functions unsuitable for further TACE. For patients who developed extrahepatic metastasis during serial TACE, treatment options including systemic targeted therapy, radiotherapy, chemotherapy, and best supportive care with or without concurrent TACE were determined again by dedicated physicians (45).

### Definition and statistical analysis

Data was presented as either mean ± standard deviation (SD) for normally distributed data or median (interquartile range (IQR)) for nonparametric ones. Categorical variables were compared by chi-squared test, while continuous variables were analyzed by the Student’s t-test or Mann Whitney U test. Overall survival (OS) was defined as the time interval between first session of TACE and last visit or death and was analyzed by the Kaplan–Meier analysis and log rank test. Cox regression multivariate analysis was used to identify independent variables that were associated with overall survival. The regression coefficients B of the independent variables were then multiplied by three and rounded to integer to generate our new prognostic scoring system. The survival of patients with different scores was assessed.

Our newly developed prognostic model was further compared with other major scoring system including BCLC stage, Six-and-Twelve prognostic score, Up-to-Seven score, AJCC stage, ALBI grade, HAP score and ART score. Both the Six-and-Twelve and Up-to-Seven scores were determined by the sum of largest tumor diameter (cm) and tumor number (13–15, 46). The ALBI score was calculated by the equation of (log10 bilirubin x 0.66) + (albumin x -0.085), where bilirubin was in μmol/L and albumin in g/L. The cut points were as follows: ≤ -2.60 (ALBI grade 1), -2.60 to ≤1.39 (ALBI grade 2), and > -1.39 (ALBI grade 3) (47, 48). The HAP score was defined as the sum of four factors including albumin < 36 g/dl, AFP > 400 ng/ml, bilirubin > 17 μmol/l and maximum tumor diameter >7 cm, with one point for each factor. The HAP score was classified as HAP-A for 0 point, HAP-B for 1 point, HAP-C for 2 points, and HAP-D for more than 2 points (11). The ART score, on the other hand, was based on three negative factors including the absence of radiologic response, the increase of aspartate aminotransferase (AST) by >25%, and an increase of Child-Pugh score of 1 after the first TACE and was further categorized into two groups (0-1.5 points and ≥ 2.5 points) (12). The discriminating efficacy of these models was examined by the receiving operating characteristics (ROC) curves and C-statistics. A 5-fold cross-validation with 100 repeats as internal validation tests were also carried out.The statistical analysis was performed with IBM SPSS Statistics 21 (IBM Corporation, Software Group, Somers, NY USA) and STATA (StataCorp. 2019. Stata Statistical Software: Release 16. College Station, TX: StataCorp LLC.). A *p*- value less than 0.05 was considered statistically significant.

## Results

### Patient characteristics

Among 260 patients enrolled, the median age was 64 years old and 192 patients (73.8%) were male. HBV infection was documented in 141 patients (54.2%) while 110 (42.3%) had chronic HCV infection. More than 60% of patients had liver cirrhosis, and the median alpha-fetoprotein (AFP) was 43.4 ng/ml. The majority of the patients (86%) were Child-Pugh classification A, and more than 70% was designated as AJCC stage I or II. The median tumor diameter was 3.3cm (range 0-7.4 cm), and 183 patients (70.4%) had multiple tumors. Bilobar involvement was the most common pattern (61.2%), followed by right side (30.0%) and left side liver lesions (8.8%). Portal vein tumor thrombus was identified in 28 patients (10.8%), with VP1/2 in 13 and VP3/4 in 15. After assessing the psoas muscle area at the level of the inferior endplate of the third lumbar vertebrae, one hundred and thirty patients (50%) were classified as sarcopenia before the first TACE. The median OS was 21.0 months for the entire cohort.

As for the major staging/scoring systems, more than 57% of the patients were classified as ALBI grade 2 and 36% as ALBI grade 1. One hundred and eleven patients (42.7%) were designated as BCLC stage C, while stage B and A accounted for 30% and 20.4% of the patients, respectively. HAP-B was the most common class (35.5%), followed by HAP-A (27.8%) and C (26.6%). ART scores of 0-1.5 points accounted for 62.4% of the patients, and 88 patients (37.6%) had scores >2.5. The demographic data and baseline characteristics were summarized in **Table 1**.

**Table 1:** Patient characteristics.

Variables	n = 260	Variables	n = 260
**Age**	64.0 (18.0)	**Tumor number**	
**Gender (male (%))**	192 (73.8)	Multiple (%)	183 (70.4)
**BMI (kg/m2)**	24.15 (4.9)	**Lobe**	
**TACE session**	4 (5)	Right (%)	78 (30.0)
**DM (yes(%))**	77 (29.6)	Left (%)	23 (8.8)
**ESRD (yes(%))**	11 (4.2)	Bilobar (%)	159 (61.2)
**Comorbidity (yes(%))**	138 (53.1)	**ALBI grade**	
**Platelet count (x 1000/uL)**	129.5 (106.25)	1 (%)	94 (36.3)
**Bilirubin (mg/dL)**	0.8 (0.5)	2 (%)	148 (57.1)
**AST (U/L)**	50.0 (47.0)	3 (%)	17 (6.6)
**Albumin (g/dL)**	3.7 ± 0.6	**Child-Pugh classification**	
**PT INR**	1.2 (0.1)	A (%)	221 (86.3)
**ICG-15 (%)**	12.73 ± 16.52 (0- 29.25)	B (%)	32 (12.5)
**Cr (mg/dL)**	0.79 (0.35)	C (%)	3 (1.2)
**AFP (ng/mL)**	43.4 (649.7)	**BCLC**	
**Cirrhosis (yes(%))**	170 (65.4)	0 (%)	8 (3.1)
**Ascites (yes(%))**	36 (13.8)	A (%)	53 (20.4)
**HBV (yes(%))**	141 (54.2)	B (%)	78 (30.0)
**HCV (yes(%))**	110 (42.3)	C (%)	111 (42.7)
**Sarcopenia (yes(%))**	130 (50.0)	D (%)	10 (3.8)
**Maximal tumor diameter(cm)**	3.3 (4.1)	**HAP score**	
**Portal vein thrombosis (yes(%))**	28 (10.8)	A (%)	69 (27.8)
**VP 1 and VP2 (yes(%))**	13 (5.3)	B (%)	88 (35.5)
**VP3 and VP4 (yes(%))**	15 (5.8)	C (%)	66 (26.6)
**AJCC stage**		D (%)	25 (10.1)
**I/II (%)**	184 (70.8)	**ART score**	
**III (%)**	76 (29.2)	0-1.5 points	146 (62.4)
		>2.5 points	88 (37.6)

AFP, alpha-fetoprotein; ALBI grade, albumin-bilirubin grade; AST, aspartate aminotransferase; ART score, Assessment for Retreatment with Transarterial Chemoembolization score; BCLC, Barcelona Clinic Liver Cancer; BMI, body mass index; DM, diabetes mellitus; ESRD, end-stage renal disease; HAP score, hepatoma arterial-embolization prognostic score; HBV, hepatitis B virus infection; HCV, hepatitis C virus infection; ICG-15, indocyanine green retention at 15 minutes; INR, international normalized ratio; PT, prothrombin time.

### Clinical features of HCC patients with sarcopenia

Compared with patients without sarcopenia, sarcopenic HCC patients were older with a higher male tendency and a significantly lower body mass index (BMI). The underlying comorbitidies and liver biochemistry were comparable between sarcopenic and non-sarcopenic groups. Surprisingly, the nutritional index albumin was not different between the two groups. As for the tumor factors, tumors in the sarcopenic group were significantly larger (median tumor diameter 3.5 vs.2.85cm, P=0.019) with a higher tumor burden (multiple tumors: 76.2% vs. 64.6%, P=0.042). The incidence of venous thrombosis was similar between the two groups. While the tumors in the sarcopenic group were more advanced according to the AJCC staging system, the disease severity based on the BCLC system was comparable between the two groups. Sarcopenic patients also had worse prognostic scores based on either ALBI grade, HAP score, or ART score (**Table 2**).

**Table 2:** Clinical characteristics of HCC patients with or without sarcopenia.

Variables	Non-sarcopenia(n = 130)	Sarcopenia(n = 130)	*P* value	Variables	Non-sarcopenia(n = 130)	Sarcopenia(n = 130)	*P* value
**Age**	62.0 (16.25)	67.5 (19.0)	0.016	**Venous thrombosis (yes(%))**	11 (8.5)	17 (13.1)	0.230
**Gender (male(%))**	89 (68.5)	103 (79.2)	0.048	**VP1 and VP2 (yes(%))**	6 (4.6)	7 (5.4)	0.718
**BMI (kg/m2)**	25.3(5.93)	23.15(4.36)	<0.001	**VP3 and VP4 (yes(%))**	5 (3.8)	10 (7.7)	0.184
**DM (yes(%))**	36 (27.7)	41 (31.5)	0.497	**ALBI grade**			0.002
**ESRD (yes(%))**	6 (4.6)	5 (3.8)	0.748	1 (%)	61 (46.9)	33 (25.6)	
**Comorbidity (yes(%))**	72 (55.4)	66 (50.8)	0.456	2 (%)	61 (46.9)	87 (67.4)	
**Platelet count(x 1000/uL)**	113.0(91.5)	139.0(117.75)	0.042	3 (%)	8 (6.2)	9 (7.0)	
**Bilirubin (mg/dL)**	0.8 (0.45)	0.8 (0.53)	0.436	**AJCC stage**			0.032
**AST (U/L)**	48.5 (41.5)	56.0 (47.5)	0.171	I/II (%)	100 (76.9)	84 (64.6)	
**Albumin (g/dL)**	3.8 ± 0.6	3.5 ± 0.6	0.119	III (%)	30 (23.1)	46 (35.4)	
**PT INR**	1.2 (0.1)	1.2 (0.1)	0.865	**Child-Pugh classification**			0.432
**ICG-15 (%)**	11.9 ± 9.3	16.2 ± 10.0	0.151	A (%)	114 (89.1)	107 (83.6)	
**Cr (mg/dL)**	0.82(0.35)	0.78(0.35)	0.744	B (%)	13 (10.2)	19 (14.8)	
**AFP ^d^ (ng/ml)**	32.9(269.9)	58.3 (1347.2)	0.054	C (%)	1 (0.8)	2 (1.6)	
**Cirrhosis (yes(%))**	87 (66.9)	83 (63.8)	0.602	**BCLC**			0.938
**Ascites (yes(%))**	14 (10.8)	22 (16.9)	0.151	0 (%)	3 (2.3)	5 (3.8)	
**HBV (yes(%))**	78 (60.0)	63 (48.5)	0.062	A (%)	28 (21.5)	25 (19.2)	
**HCV (yes(%))**	50 (38.5)	60 (46.2)	0.209	B (%)	40 (30.8)	38 (29.2)	
**Maximal tumor diameter(cm)**	2.85 (3.85)	3.5 (5.15)	0.019	C (%)	54 (41.5)	57 (43.8)	
**Tumor number**			0.042	D (%)	5 (3.8)	5 (3.8)	
**Single (%)**	46 (35.4)	31 (23.8)		**HAP score**			<0.001
**Multiple (%)**	84 (64.6)	99 (76.2)		A (%)	49 (39.2)	20 (16.3)	
**Lobe**			0.719	B (%)	46 (36.8)	42 (34.1)	
**Right (%)**	41 (31.5)	37 (28.5)		C (%)	23 (18.4)	43 (35.0)	
**Left (%)**	12 (9.2)	11 (8.5)		D (%)	7 (5.6)	18 (14.6)	
**Bilobar (%)**	77 (59.3)	82 (63.0)		**ART score**			0.084
				0-1.5 points (%)	81 (68.6)	65 (56.0)	
				>2 points (%)	37 (31.4)	51 (44.0)	

AFP, alpha-fetoprotein; ALBI grade, albumin-bilirubin grade; AST, aspartate aminotransferase; ART score, Assessment for Retreatment with Transarterial Chemoembolization score; BCLC, Barcelona Clinic Liver Cancer; BMI, body mass index; DM, diabetes mellitus; ESRD, end-stage renal disease; HAP score, hepatoma arterial-embolization prognostic score; HBV, hepatitis B virus infection; HCC, hepatocellular carcinoma; HCV, hepatitis C virus infection; ICG-15, indocyanine green retention at 15 minutes; INR, international normalized ratio; PT, prothrombin time.

### Prognostic significance of sarcopenia for overall survival in patients receiving TACE

**Figure 2:**
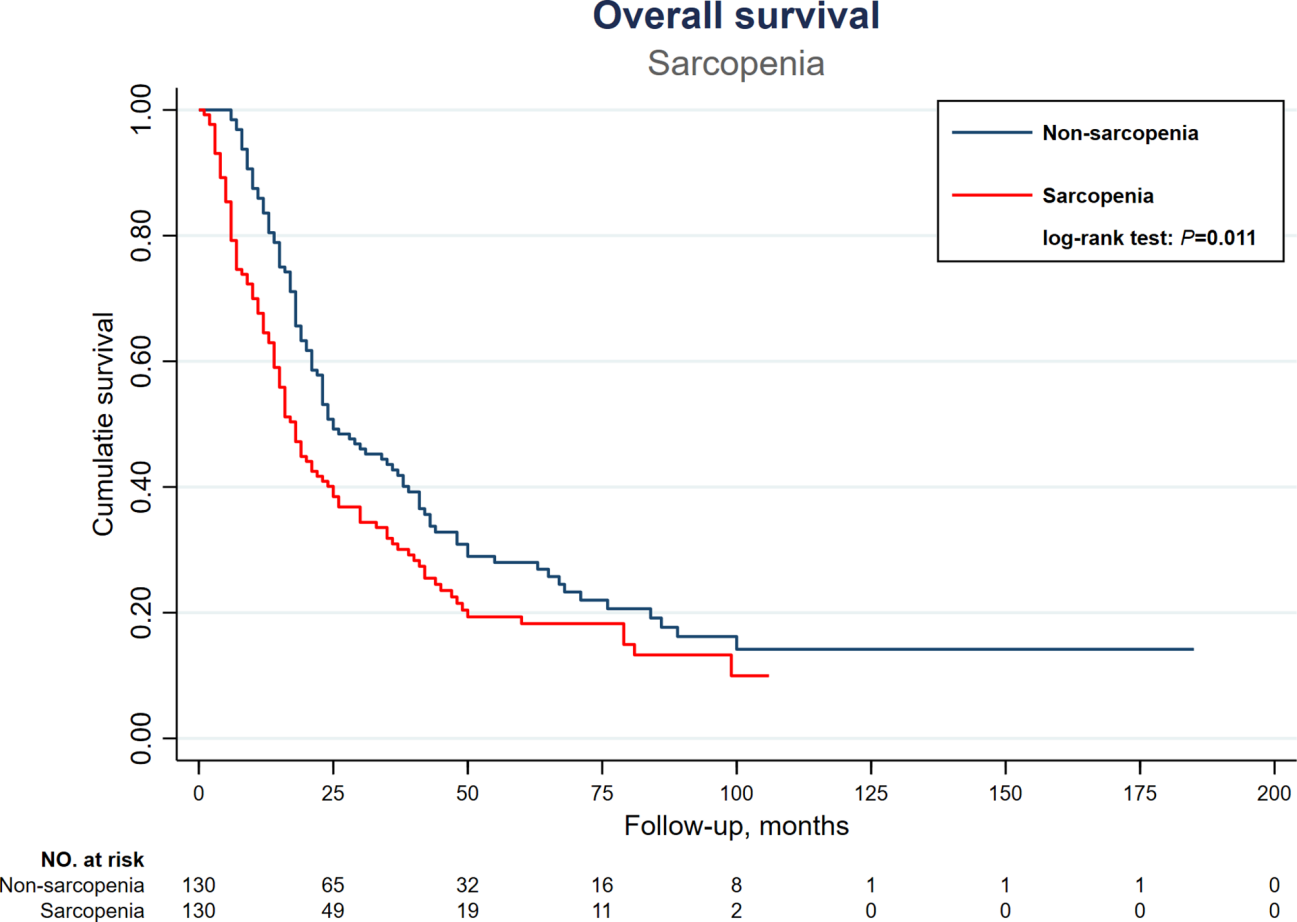
Kaplan-Meier overall survival curves of HCC patients receiving TACE therapy, stratified by sarcopenia. HCC, hepatocellular carcinoma; TACE, transarterial chemoembolization.

After univariate analysis, neither age, gender, BMI, comorbidity, viral hepatitis, cirrhosis, liver biochemistry, nor Child –Pugh classification significantly influenced OS after TACE. On the other hand, multiple tumors (P=0.006), maximal tumor diameter ≥ 5cm (P <0.001), alpha-fetoprotein ≥ 200ng/dL (P <0.001), portal venous thrombosis (P=0.002), major venous thrombosis (VP3 and VP4, P<0.001), and albumin <3.5mg/dL(P=0.054) were found to be significantly associated with a worse OS. Moreover, sarcopenia also significantly impaired OS in HCC patients receiving TACE (18 vs. 25 months, P= 0.011) (**Figure 2**). The major scoring systems were also prognostic of OS in HCC after TACE (all P <0.05) (**Table 3**).

**Table 3:** Univariate analysis of factors for overall survival in HCC patients receiving TACE.

Variables		n = 260	Median survival (95%CI)	*P* value	Variables		n = 260	Median survival (95%CI)	*P* value
**Age (yr)**	≧65	127	23.0 (18.8-27.2)	0.838	**ALBI grade**	1	94	34.0 (19.1-48.9)	0.014
	<65	133	21.0 (16.5-25.5)			2	148	19.0 (15.6-22.4)	
**Gender**	M	192	21.0 (17.3-24.7)	0.928		3	17	16.0 (10.6-21.4)	
	F	68	23.0 (18.7-27.3)		**BCLC**	0	8	Not reached	0.007
**BMI**	≧27	58	23.0 (17.7-28.3)	0.722		A	53	42.0 (25.4-58.6)	
	<27	200	21.0 (16.9-25.1)			B	78	20.0 (16.1-23.9)	
**Comorbidity**	Yes	138	24.0 (16.8-31.2)	0.337		C	111	18.0 (12.9-23.1)	
	No	122	18.0 (15.2-20.8)			D	19	13.0 (8.9-17.1)	
**DM**	Yes	77	23.0 (14.6-31.4)	0.609	**AFP (ng/mL)**	≧200	86	16.0 (13.0-19.0)	<0.001
	No	183	21.0 (17.7-24.3)			<200	169	30.0 (22.1-37.9)	
**ESRD**	Yes	11	10.0 (3.5-16.5)	0.087	**Tumor number**	Multiple	183	19.0 (16.0-22.0)	0.006
	No	248	23.0 (19.5-26.5)			Single	77	33.0 (22.2-43.8)	
**HBV**	Yes	141	22.0 (17.8-26.2)	0.555	**Maximal tumor diameter(cm)**	≧ 5	84	14.0 (11.6-16.4)	<0.001
	No	119	23.0 (17.3-28.7)			<5	170	30.0 (20.6-39.4)	
**HCV**	Yes	110	22.0 (17.8-26.2)	0.992	**Bilobe**	Yes	97	16.0 (12.2-19.8)	0.084
	No	150	23.0 (18.5-27.5)			No	163	24.0 (18.9-29.1)	
**Cirrhosis**	Yes	170	23.0 (19.2-26.8)	0.858	**Venous thrombosis**	Yes	28	10.0 (5.6-14.4)	0.002
	No	90	21.0 (14.6-27.4)			No	232	23.0 (18.5-27.5)	
**Ascites**	Yes	36	16.0 (11.1-20.9)	0.461	**VP1 and VP2**	Yes	13	16.0 (12.6-19.4)	0.576
	No	223	23.0 (19.5-26.5)			No	232	23.0 (18.5-27.5)	
**AST (U/L)**	≧102	37	19.0 (13.2-24.8)	0.568	**VP3 and VP4**	Yes	15	7.0 (4.8-9.2)	<0.001
	<102	220	23.0 (19.4-26.6)			No	245	23.0 (19.4-26.6)	
**Bilirubin (mg/dL)**	≧1.3	49	20.0 (13.1-26.9)	0.373	**Sarcopenia**	Yes	130	18.0 (14.9-21.1)	0.011
	<1.3	211	23.0 (18.9-27.1)			No	130	25.0 (17.2-32.8)	
**Albumin (g/dL)**	≧3.5	162	23.0 (15.9-30.1)	0.054	**HAP score**	A	69	41.0 (30.9-51.1)	<0.001
	<3.5	97	19.0 (15.1-22.9)			B	88	23.0 (18.5-27.5)	
**ICG-15**	≧10%	26	25.0 (8.7-41.3)	0.517		C	66	18.0 (14.5-21.5)	
	<10%	16	41.0 (0-82.1)			D	25	11.0 (6.1-15.9)	
**Child-Pugh classification**	A	221	23.0 (18.5-27.5)	0.315	**ART score**	0-1.5 points	146	29.0 (19.7-38.3)	0.001
	B	32	19.0 (11.2-26.8)			>2.5 points	88	17.0 (13.7-20.3)	
	C	3	15.0 (11.8-18.2)						

AFP, alpha-fetoprotein; ALBI grade, albumin-bilirubin grade; AST, aspartate aminotransferase; ART score, Assessment for Retreatment with Transarterial Chemoembolization score; BCLC, Barcelona Clinic Liver Cancer; BMI, body mass index; DM, diabetes mellitus; ESRD, end-stage renal disease; HAP score, hepatoma arterial-embolization prognostic score; HBV, hepatitis B virus infection; HCC, hepatocellular carcinoma; HCV, hepatitis C virus infection; ICG-15, indocyanine green retention at 15 minutes; INR, international normalized ratio; PT, prothrombin time; TACE, transarterial chemoembolization.

Cox regression multivariate analysis further demonstrated that multiple tumors (P=0.002), maximal tumor diameter≥ 5cm (P=0.01), major venous thrombosis (VP3 and VP4, P=0.001), sarcopenia (P=0.048), AFP ≥ 200 ng/ml (P <0.001), and albumin<3.5mg/dL(P=0.024) were independent poor prognostic factors for overall survival in HCC patients receiving TACE (**Table 4**). Patients with sarcopenia had a 1.36-fold risk of death after TACE compared to those without sarcopenia.

**Table 4:** Cox regression multivariate analysis of prognostic factors for overall survival in HCC patients receiving TACE.

		Overall survival				
Variables		HR	95% CI	B	*P* value	Score allocation
**Maximal tumor diameter ≧ 5 cm**	**Yes**	**1.543**	1.107-2.152	0.434	0.01	**1.5**
	No	1				
**Multiple tumors**	**Yes**	**1.723**	1.216-2.44	0.544	0.002	**1.5**
	No	1				
**AFP ≧ 200ng/mL**	**Yes**	**1.969**	1.428-2.714	0.677	<0.001	**2**
	No	1				
**Albumin <3.5 g/dL**	**Yes**	**1.428**	1.048-1.946	0.356	0.024	**1**
	No	1				
**VP3 and VP4**	**Yes**	**2.58**	1.466-4.54	0.948	0.001	**3**
	No	1				
**Sarcopenia**	**Yes**	**1.361**	1.003-1.848	0.309	0.048	**1**
	No	1				

AFP, alpha-fetoprotein; B, regression coefficient; CI, confidence interval; HCC, hepatocellular carcinoma; HR, hazard ratio; TACE, transarterial chemoembolization.

Values in bold under ‘HR’ denote the “relative” risk of death after TACE.

### The development of a new prognostic scoring system

**Figure 3:**
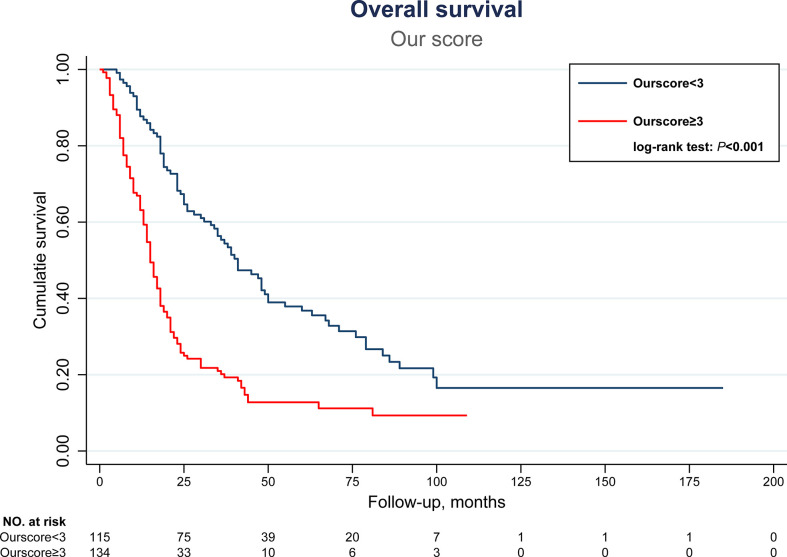
Kaplan-Meier overall survival curves of HCC patients receiving TACE therapy, stratified by our newly proposed scoring system with cutoff set at 3. HCC, hepatocellular carcinoma; TACE, transarterial chemoembolization.

Next, efforts were made to formulate a new scoring system incorporating sarcopenia. The respective calculated regression coefficients (B-value) of the 6 independent prognostic factors identified above were multiplied by 3 and rounded to integer in order to formulate a new scoring system predictive of OS after TACE. As shown in **Table 4**, multiple tumors, maximal tumor diameter ≥ 5cm, major venous thrombosis (VP3 and VP4), sarcopenia, AFP ≥ 200 ng/ml, and albumin<3.5mg/dL were allocated with a score of 1.5, 1.5, 3, 1, 2, and 1, respectively. The newly proposed scoring system, “our score”, was the summation of the scores of 6 independent factors. By performing ROC analysis, our score was found to be significantly correlated with both 3-year and 5-year OS, with higher score indicating higher risk of death (area under the ROC (AUROC) of 3-year and 5-year OS, 0.757 and 0.751, P <0.001 and <0.001, respectively) (**Table 5**). Further analysis verified that a cutoff set at 3 can classify patients into two groups with distinct prognosis. Patients with “our score” less than 3 (115 patients) can enjoy an OS of 41 months (95% CI: 32.5-49.5), in contrast to only 15 months (95% CI: 13-17) in those with a score ≥ 3 (134 patients) (P < 0.001) (**Figure 3**).

**Table 5:** The discriminative ability for overall survival of different models.

Score system	3-yr OS AUROC (95% CI)	*P* value	5-yr OS AUROC (95% CI)	*P* value
**Up to seven**	0.681 (0.593-0.768)	<0.001	0.637 (0.533-0.741)	0.014
**6 and 12**	0.681 (0.593-0.768)	<0.001	0.637 (0.533-0.741)	0.014
**BCLC**	0.604 (0.509-0.698)	0.037	0.618 (0.513-0.723)	0.034
**AJCC TNM stage**	0.650 (0.562-0.738)	0.002	0.654 (0.557-0.752)	0.006
**ART score**	0.668 (0.576-0.759)	0.001	0.634 (0.528-0.740)	0.016
**ALBI grade**	0.562 (0.462-0.662)	0.212	0.593 (0.479-0.706)	0.097
**HAP score**	0.658 (0.568-0.748)	0.001	0.676 (0.583-0.770)	0.002
**Our score**	0.757 (0.679-0.836)	<0.001	0.751 (0.662-0.84)	<0.001

ALBI grade, albumin-bilirubin grade; AJCC, American Joint Committee on Cancer; ART score, Assessment for Retreatment with Transarterial Chemoembolization score; AUROC, area under the receiver operating characteristics; BCLC, Barcelona Clinic Liver Cancer; HAP, hepatoma arterial embolization prognostic score; OS, overall survival.

**Figure 4:**
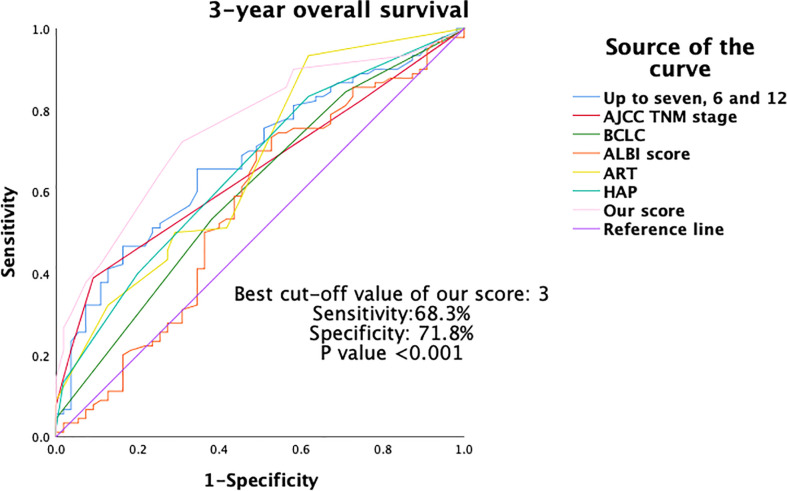
Area under the ROC (AUROC) of 3-year OS of different scoring systems. OS, overall survival; ROC, receiver operating characteristics.

**Figure 5:**
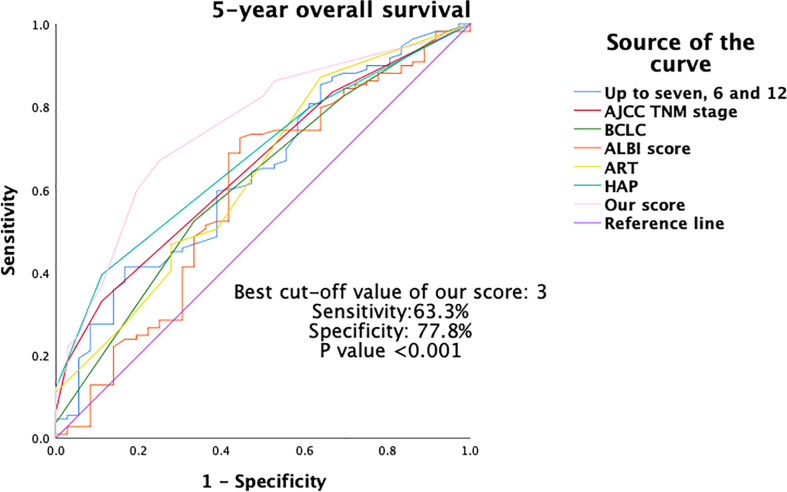
Area under the ROC (AUROC) of 5-year OS of different scoring systems. OS, overall survival; ROC, receiver operating characteristics.

**Figure 6:**
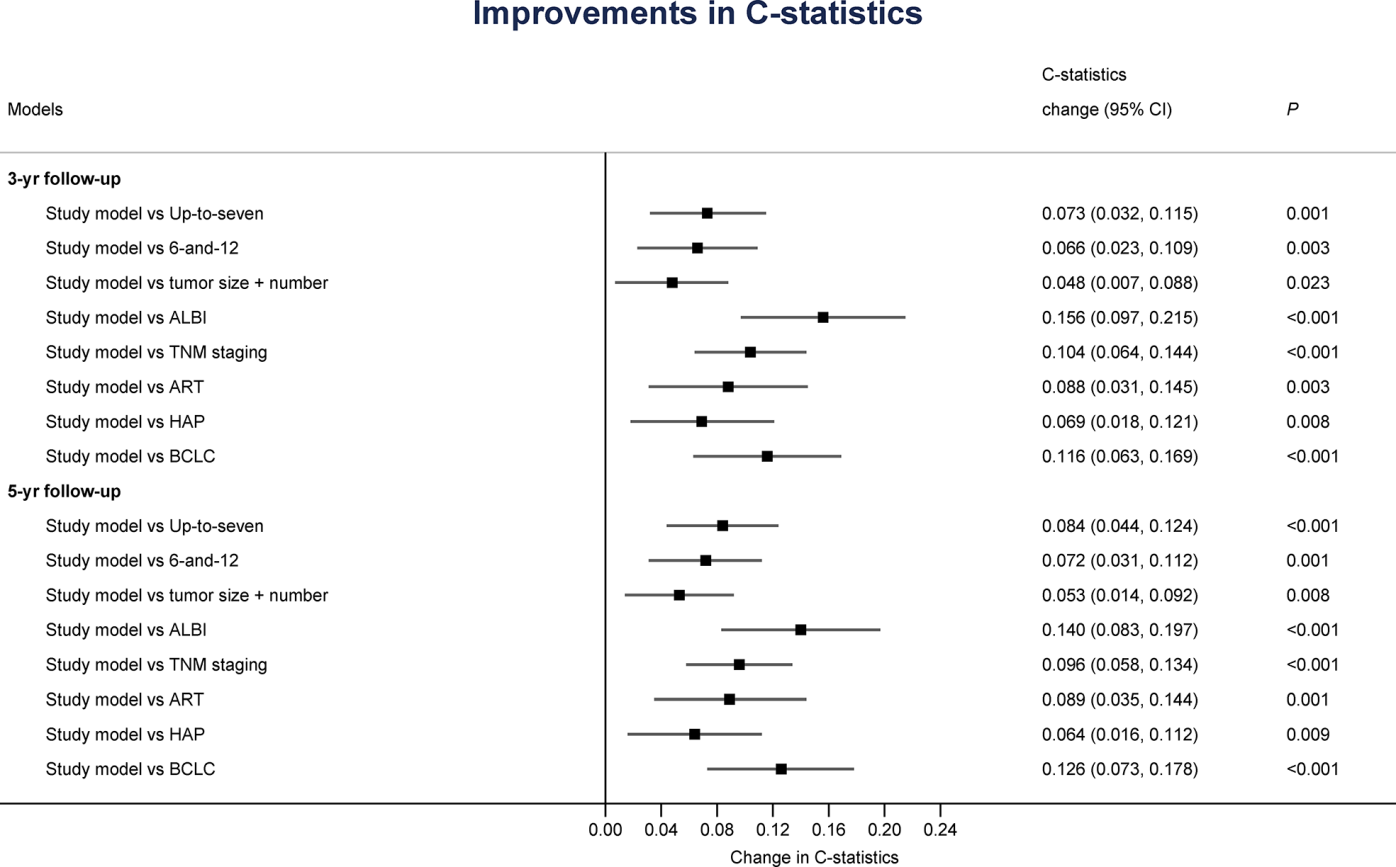
Improvements of the current scoring system in discriminative ability for overall survival as analyzed by C-statistics.

Furthermore, the discriminative ability for OS was compared between the current new model and other major scoring systems by AUROC and C-statistics. As shown in **Table 5**, and **Figures 4**, **5**, our score had the highest AUROC for both 3-year and 5- year OS after TACE. A 5-fold cross-validation with 100 repeats also confirmed our findings. C-statistics further demonstrated significant improvements of the current study model as compared to other scoring systems for the prediction of OS (all P <0.05) (**Figure 6**).

## Discussion

In patients who undergo major surgery, sarcopenia is associated with worse outcomes including post-surgical complications, short-term survival, and long-term results (18, 49–53). Our recent study further identified that sarcopenia was an independent predictive factor for ventilator weaning and ICU mortality among critically ill patients after major abdominal operations (54). In addition, sarcopenia has also been an unfavorable prognostic factor for patients with cirrhosis and HCC (34). The impact of sarcopenia on the prognosis of HCC undergoing TACE, on the other hand, was still under debate. There were studies showing that baseline muscle volume mass was not associated with clinical outcome in HCC receiving TACE (34, 55, 56). In contrast, there were other studies revealing that pre-interventional sarcopenia was an independent predictor for an unfavorable outcome (34, 35, 57, 58). The conflicting results may be derived from their limited sample size and heterogeneous patient population. Our study, which comprised 260 HCC patients, is by far one of the largest series in the English literature to document the negative influence of baseline sarcopenia on the survival of HCC patients undergoing TACE.

Sarcopenia, as an independent poor prognostic factor for HCC patients receiving TACE therapy, was found to be significantly associated with an older age, male tendency, and lower BMI. Our findings were mostly consistent with the study done by Nasimi et al. that older age and low BMI were associated with a higher risk of low skeletal muscle mass (59). However, unlike their result, the current study did not find significant association between serum albumin and sarcopenia. This can be partly explained by the fact that more than 65% of HCC patients, either sarcopenic or not, had cirrhosis. The impaired albumin production imposed by liver dysfunction may thus mask the association between albumin and sarcopenia. Our findings thus implicated that serum albumin, which is generally considered to be a nutritional marker, cannot objectively reflect the general status of the body composition in patients with HCC. Skeletal muscle mass or sarcopenia, on the other hand, may be a more appropriate surrogate to represent the general status or nutritional condition in the context of HCC.

Furthermore, the current study also found that HCC in sarcopenic patients were more advanced in terms of tumor size, tumor numbers, and AJCC tumor stage. The sarcopenic patients also had worse prognostic scores. The causal relationship between sarcopenia and disease severity requires further investigations. In the meanwhile, we can only conclude that in HCC patients receiving TACE therapy, sarcopenia is associated with significantly reduced long-term survival. Stringent patient selection is thus warranted to optimize patient survival after TACE. Radiotherapy or systemic therapy with either tyrosine kinase inhibitors or checkpoint inhibitors, along with TACE or not, should be considered for sarcopenic patients with unresectable HCC (2, 3). Further prospective studies are warranted to validate our findings.

To further stratify HCC patients indicated for TACE, we developed a scoring system which incorporated independent factors prognostic for OS. In addition to sarcopenia, our newly proposed scoring system also included factors such as multiple tumors, maximal tumor diameter≥ 5cm, major venous thrombosis, AFP ≥ 200 ng/ml, and albumin<3.5mg/dL. Although most of the variables included in the current study model, except for sarcopenia, were well-known prognostic factors for HCC, they were endowed with a specific score to represent their impact on the OS. For example, venous thrombosis at VP3/VP4 was found to be one of the most dismal prognostic factors for HCC receiving TACE (Score 3). In other words, patients with major venous thrombosis may not be optimal candidates for TACE. This was consistent with major treatment guidelines worldwide in which systemic therapy should be considered for HCC with portal invasion (2). The current scoring system also possessed other advantages. First, unlike other scores which require logarithmic calculations, our score was simply the summation of six individual scores (10). Second, the product of our score could be obtained before the administration of first TACE. We did not have to evaluate the treatment response or liver damage resulting from the first TACE (12). Next, our scoring system considered both tumor (number, size, AFP, major venous thrombosis), liver (albumin), and patient factors (sarcopenia) at the same time. Although BCLC stage also includes patient performance status, sarcopenia was more objectively assessed. This may explain why our predictive capability in terms of AUROC was significantly better than other major scoring systems. The higher our score is, the shorter the OS can be achieved if TACE is going to be administered. As a result, we should consider either combination or systemic therapies for patients with higher scores (2, 3). Further large-scale well-designed prospective studies are definitely necessary to approve our hypothesis.

Despite remarkable findings, the present study still had several limitations. First, the mechanism underlying sarcopenia in HCC remains unclear. The causal relationships between sarcopenia and HCC were also elusive. Among independent prognostic factors discovered herein, sarcopenia and albumin were the only factors that can possibly be manipulated. If the mechanistic relationships cab be unraveled, we can potentially alter the dismal fates of these HCC patients. Although Shiozawa et al. had discovered that early supplement of branched-chain amino acids or L-carnitine-enriched nutrients may improve the treatment outcome of HCC, the relationship between these metabolites and sarcopenia is still uncertain and deserves further investigations (60, 61). Second, the retrospective nature rendered selection bias inevitable. Third, the lack of external validation cohorts also made our findings less convincing. Last but not the least, the current study only identified HCC patients with sarcopenia or higher prognostic scores were likely to have worse survival after TACE. We haven’t provided any validated treatment alternatives for these patients at risk. Future multi-center prospective studies comprising different treatment modalities are warranted to establish appropriate treatment suggestions.

## Conclusion

In conclusion, our study demonstrated that sarcopenia was an independent prognostic factor for HCC undergoing TACE therapy. Our newly developed score model, which incorporated, in addition to patient factor sarcopenia, tumor number, tumor size, major venous thrombosis, serum AFP, and albumin into consideration, could effectively predict patient survival after TACE. Physicians could, based on the current score model, carefully select candidate patients for TACE treatment in order to optimize their survival. In contrast, since HCC patients with sarcopenia or higher prognostic scores may not benefit from TACE, future multi-center prospective studies comprising different treatment modalities are warranted to establish appropriate treatment suggestions for them.

## Data availability statement

The dataset will be available only under the approval of Chang Gung Memorial Hospital. Requests to access the datasets should be directed to alanchaoweilee@hotmail.com.

## Ethics statement

This study was approved by the Institutional Review Boards (CGMH IRB No: 202102599B0) of CGMH. Written informed consent for participation was not required for this study in accordance with the national legislation and the institutional requirements.

## Author contributions

T-PC and S-FH designed the study, conducted the research, and drafted the manuscript. They contributed equally to this work; W-HC and K-TP performed the transarterial chemoembolization, formulate the concept, and revised the manuscript. M-CY and W-CL confirmed the analysis, supervised the study, and revised the manuscript; H-IT, P-TL, and J-HC collected the data, interpreted the results, and revised the manuscript; H-YC performed the statistics and analyzed the results; C-WL coordinated the entire study, performed the statistics, and approved the manuscript; All authors read and approved the final manuscript.

## Funding

This study was supported by Chang Gung Memorial Hospital (CMRPG3L1641, CMRPG3L1831, and CORPG3J0571).

## Acknowledgments

Appreciation is especially paid to our colleges in the Department of Cancer Center, Department of Medical Imaging and Intervention, Department of Gastroenterology and Hepatology, Linkou Chang Gung Memorial Hospital and Graduate Institute of Clinical Medical Sciences, Chang Gung University for their technical support.

## Conflict of interest

The authors declare that the research was conducted in the absence of any commercial or financial relationships that could be construed as a potential conflict of interest.

## Publisher’s note

All claims expressed in this article are solely those of the authors and do not necessarily represent those of their affiliated organizations, or those of the publisher, the editors and the reviewers. Any product that may be evaluated in this article, or claim that may be made by its manufacturer, is not guaranteed or endorsed by the publisher.

## Abbreviations

AFP, alpha-fetoprotein; ALBI grade, albumin-bilirubin grade; ART score, Assessment for Retreatment with Transarterial Chemoembolization score; AUROC, area under receiver operating characteristic curve; BCLC, Barcelona Clinical Liver Cancer; BMI, body mass index; CI, confidence interval; CLIP, the Cancer of the Liver Italian Program; CT, computed tomography; DM, diabetes mellitus; ESRD, end-stage renal disease; HAP score, Hepatoma arterial-embolization prognostic score; HBV, hepatitis B virus infection; HCC, hepatocellular carcinoma; HCV, hepatitis C virus infection; HR, hazard ratio; IQR, interquartile range; RFA, radiofrequency ablation; ROC, receiver operating characteristic; TACE, transarterial chemoembolization; TNM, Tumor-Node-Metastasis.
